# Nutrition and Health Programming and Outreach in Grocery Retail Settings: A Community Coalition in Action

**DOI:** 10.3390/nu15040895

**Published:** 2023-02-10

**Authors:** Dennis Ogeto Nyachoti, Alisha H. Redelfs, Louis D. Brown, Eufemia B. Garcia, Erica Garcia, Carlos A. Loweree, Karen Del Rio, Daniel Del Toro, Denise Vasquez, Gabriela A. Gallegos, Michael P. Kelly, Leah D. Whigham

**Affiliations:** 1 Center for Community Health Impact, University of Texas Health Science Center School of Public Health, El Paso Campus, El Paso, TX 79905, USA; 2 Department of Public Health, Brigham Young University, Provo, UT 84602, USA; 3 Colonias Program, Department of Engagement for Sustainability, Division of Academic and Strategic Collaborations, Socorro, TX 79927, USA; 4 WIC Program, City of El Paso Department of Public Health, El Paso, TX 79905, USA; 5 Food City Supermarkets, El Paso, TX 79905, USA; 6 Teaching Learning and Culture Program, University of Texas at El Paso, El Paso, TX 79968, USA; 7 Centro San Vicente Health Center, El Paso, TX 79915, USA; 8 Paso del Norte Health Foundation, El Paso, TX 79901, USA

**Keywords:** partnership, collaboration, grocery stores, coalition, access, healthy food, community nutrition

## Abstract

Grocery stores can provide a conducive environment for interventions targeting healthy eating and access to health services, particularly in low-income communities. A wide array of organizations deliver nutrition and related programs in community settings, but rarely in a coordinated fashion. Collaboration of local health promotion organizations with grocery stores could increase consumers’ access to and selection of healthy foods and related services. This evaluation of the In-Store Programming and Outreach Coalition (IPOC) uses thematic analysis of first-person accounts from coalition members. To our knowledge, this is the first study of such a coalition. We present perspectives from six stakeholders about the IPOC strengths, challenges, and recommendations for strengthening the delivery of in-store interventions. Themes identified include partnership, increased client reach and cross-referrals, conflicting work schedules, leadership, and recommendations to identify coalition leaders and expand services to other grocery stores. We conclude that grocery stores can offer a suitable setting for programming and community outreach through coalitions.

## 1. Introduction

In the United States, about 10.9% of the population had food insecurity in 2019, with a higher rate of 14.6% in El Paso, TX [1]. The federal Household Pulse Survey deployed during the COVID-19 pandemic estimated national levels of food insufficiency peaked at over 13% in 21 December 2020, and was just under 11% as of November 2022 [2]. Ethnic minorities experience higher rates of food insecurity. Over 23% of the Hispanic population lacks adequate and consistent food access [3] They are also four times more likely to lack access to healthy food choices than the general public [4,5]. Food insecurity and access to healthy and affordable food options are separate but interrelated problems. Increased food insecurity in the US is attributed to lack of access to healthy, safe, and nutritious food due to lack of money [3].

Grocery stores can provide a conducive environment to increase access and availability of healthy food choices, particularly in low-income communities [6,7]. Philadelphia, Pennsylvania, is one of several locations that have implemented grocery store interventions from policy, systems, and environmental changes perspectives and has shown promising results [8,9]. Other regions have also offered federally funded health and nutrition programs at grocery stores, with evaluations showing increased access to the programs [10].

Grocery stores are central gathering points for many communities. Nationally, numerous grocery store interventions in the public health field have sought to increase access to healthy food and change dietary behaviors among customers (among others, see [11,12,13,14]. However, findings from grocery store interventions using collaborative efforts have shown mixed or limited evidence of impact on health behaviors [15,16]. Coordinated program delivery, with the pooling of resources, talents, and strategies from multiple sectors, may effectively impact population health and well-being [17]. Additionally, there are inherent challenges in collaborating with grocery stores since, as for-profit entities, they understandably need to prioritize profits for survival.

In 2017, under the coordination of the Paso del Norte Institute for Healthy Living (now called the UTHealth Center for Community Health Impact, or CCHI), a community assessment that focused on access to healthy affordable food was conducted in El Paso County, Texas. The assessment used key informant interviews, community member surveys, and spatial identification of high-need areas using GPS maps of grocery store sales, income level, and death due to diet-related diseases [18]. Regional data along with national best practices for addressing access to healthy and affordable food were presented at a stakeholder convening called the Paso del Norte Food Summit. Input from stakeholders led to three key priority areas: increase participation in SNAP, develop partnerships with food retailers, and develop a healthy food financing initiative [18]. Development of partnerships with food retailers was accomplished through the coalition-based strategy described here through establishing the In-Store Programming and Outreach Coalition (IPOC).

### Coalition Approach

A coalition is “a group of individuals representing diverse organizations, factions, or constituencies within the community who agree to work together to achieve a common goal” [19]. A coalition is formed when different sectors and organizations are merged to achieve a collective purpose, goal, or agenda [20,21]. Health promotion coalitions are built on the assumption that minimal success could be achieved when the interventions are implemented separately compared to a partnership approach [22].

The Community Coalition Action Theory (CCAT) [19,23] guided the IPOC design and implementation. CCAT consists of three stages: formation, maintenance, and institutionalization [19]. In the formation stage, a lead agency invites other partners with similar goals and aspirations to form a partnership. In the case of IPOC, the CCHI followed up with stakeholders from the Food Summit who voiced an interest in food retail-based program delivery and outreach. Additional CCHI partners were contacted who had shared interests. Interested partners who had the capacity to deliver health-related programming and outreach formed the coalition, and operations and activities were initiated (CCAT maintenance stage). Other coalition components such as synergy and pooling of resources occur during the maintenance stage, informing community change (institutionalization stage) [19]. This evaluation details how these components came together for IPOC.

Many studies have assessed the effectiveness of coalitions to deliver health services [24,25], but questions on their generalizability have been raised, especially with finding an appropriate and unified evaluation methodology [26]. There is also limited literature on stakeholders’ views on coalition development and operationalization. We are not aware of any studies documenting the challenges of coalitions that focus on food retail locations for delivery of programs and outreach. Thus, there is a need for stakeholder input, reflection, and thoughts about the IPOC coalition, specifically related to the unique involvement of food retail partners. This was a unique opportunity for stakeholders to be heard regarding their experiences with the coalition and suggest recommendations. This paper aims to inform community practice-based partnerships and innovative grocery store interventions to increase access to health programming and services. To our knowledge, this is the first study of a collaborative in-store coalition in the US-Mexico border and the nation. Specifically, this case study examined the strengths and challenges of a coalition involving grocery store partners through input from partners coordinating and implementing the IPOC. In addition to identifying the coalition’s strengths and challenges, the evaluation team identified recommendations from the stakeholder narratives for improving service delivery. IPOC stakeholders produced first-person accounts of the coalition’s work focusing on strengths, challenges, and recommendations for the IPOC. We review and identify themes from these accounts and propose innovative approaches to address challenges and increase the overall effectiveness and sustainability of the IPOC. We anticipate our experiences can serve as a guide for those interested in working with grocery stores using a community coalition model.

The IPOC is slated for expansion to sites funded by El Paso County’s Healthy Food Financing Initiative (HFFI, another priority outcome from the Paso del Norte Food Summit), which invests in economic development focused on food retail to expand the availability and affordability of healthy food. The IPOC was integrated into the HFFI policy; grantees agree to make space available and host IPOC services.

## 2. Materials and Methods

### 2.1. The IPOC (Coalition)

The IPOC’s purpose was multi-faceted; the coalition aimed to collaboratively address food insecurity, nutrition education, and access to healthcare and healthy, affordable food in El Paso County. The founding coalition members are described in Table 1 along with their contribution to the in-store program delivery or outreach. All three Food City locations primarily serve low-income Hispanic clientele. The coalition members held regular meetings to plan, coordinate, and provide updates on their implementation progress.

### 2.2. The IPOC (Intervention)

Within the three grocery stores, the coalition partners coordinated simultaneous delivery of programs and outreach in order to cross-refer shoppers to the different programs and increase the exposure of shoppers to services from all coalition partners. Coalition partners also communicated to ensure similar services were not overlapping simultaneously at a single location. Each week, the following would be offered: Heart Smarts, 8 lessons to educate shoppers on healthy eating choices (AgriLife or the Colonias Program), WIC services, including enrollment support and eligibility recertification (El Paso Department of Public Health), SNAP outreach enrollment assistance (Central San Vicente), and health screenings, including measurement of height, weight, blood pressure, and chronic disease risk screening (UTEP or UTHealth). Store owners provided space to set up tables for all coalition activities. Service delivery timing and frequency was coordinated among coalition member and typically happened on a weekday with weekly or biweekly frequency. This collaboration with grocery stores aimed to increase consumers’ access to and selection of healthy foods and healthcare services.

### 2.3. Evaluation: First-Person Accounts

The IPOC decided to conduct an evaluation using first-person accounts to encourage reflection on the coalition and how it has impacted their organizational goals, including but not limited to identifying the coalition’s strengths and challenges [25,27]. Such narratives can inform policy development and provide opportunities to connect with broader social groups and populations represented by narrators [28].

Following protocol approval by the Institutional Review Boards of UTHealth School of Public Health (primary) and the University of Texas at El Paso, a total of 10 coalition members who participated in grocery store interventions (WIC, SNAP, Heart Smart, and health screenings) and store owners or their designee consented to participate. All 10 completed written first-person accounts, but only six narratives met the described purpose of the study (some partners only reported how the community had received their interventions and not the impact on their organization or reflections on the coalition). However, there was at least one narrative from each organization in the coalition that met the evaluation goals. Narrative prompts are included in Table 2.

The evaluation team from UTHealth CCHI compiled and edited responses for readability, and allowed the partners to review and approve the narratives. Next, each meaning unit was matched to the respective research question, and themes were identified by the evaluation team [29]. Narratives were coded by two reviewers based on themes. Any differences in coding were discussed and resolved. Finally, the evaluators reached a consensus on the most suitable themes for the discussion section [29] and conducted a cross-case analysis.

## 3. Results

### 3.1. First-Person Account #1: Manager, Food City Supermarkets

The program delivery by the in-store coalition members in our stores has a positive impact on our customers. Some of the activities conducted, such as offering shoppers demonstrations of recipes that they can make at home has been beneficial. It has also impacted our stores positively because the recipes feature ingredients in our weekly advertisement special. In most cases, we see an increase in sales of items used in the recipe demonstrations, which is a good thing for our business too. One challenge we have faced is that some recipes do not resonate with the older customers. If nutrition education coalition partners could identify recipes that better meet the needs of this specific clientele, that would be useful.

One thing that could be improved upon is to have better coordination between the demonstration recipe of the week and the displays we set up in the store to ensure ingredients for the recipe are prominently displayed, so customers know how to find it. We would also be willing to offer pricing incentives that align with the ingredients featured in that week’s recipe.

Having seen how the in-store coalition is impacting our customers, who are primarily low-income, we hope the coalition will continue working in our grocery stores to reach low-income members of our community.

### 3.2. First-Person Account #2: Coordinator, Heart Smarts Colonias Program

We joined the coalition and its partners because of the opportunities to enhance the nutrition education services we already offer in the grocery stores. We wanted to create a healthier community where we could provide free services to improve the health or well-being of people. The objective of the coalition is to offer complementary health related services for the consumer. Having other coalition members to provide input and feedback on possible improvements or problem solving has also been an advantage of coalition partnership. Participation in this coalition has increased our understanding of integral operations of grocery stores as well as the Healthy Food Financing Initiative. I was able to directly observe the benefits of coordination of coalition members. For example, I witnessed several customers take the opportunity to consult with the WIC representative about eligibility and benefit questions, for which they received immediate guidance.

The coalition has been effective in networking and coordinating many diverse organizations to deliver programs and outreach simultaneously. However, even greater coordination is needed, especially when changes within organizations, such as personnel, can deter progress somewhat. In addition, it is often challenging to coordinate with store managers. This could be due to time pressures on store managers and, in the case of some stores, a lack of understanding of the programming delivered and the value it can bring to the stores and their customers.

Some practical suggestions for increasing coordination and impact include inclusion of the recipe of the week in the store flier, better signage within the store directing shoppers to the coalition activities, and feedback to store managers regarding the numbers and types of customers engaged so they can better see the impact of the programming. In addition, it would be useful to have a brief document outlining the purpose of the coalition that could be shared when approaching new stores to help reduce anxiety or mistrust in adopting this kind of programming in their stores. There are many stores that are interested in hosting the coalition partners, so it is important that we find ways to expand our capacity.

### 3.3. First-Person Account #3: Public Health Specialist, WIC Program, City of El Paso Public Health Department

I decided to participate in the in-store coalition to increase awareness of our program out in the community. One of the biggest advantages of being part of this has been creating a partnership with other organizations. Also, since joining the coalition we have seen an increase of participation in our program in the grocery stores. The coalition has positively impacted our program by increasing awareness of our program. We have also been able to build stronger partnerships with the community and collaborating agencies in this coalition.

There is a difference when we implement our program separately from the coordinated effort of the coalition. The services we offer are a little more limited, so the customers benefit from the coordinated delivery of programming and outreach offered by other coalition members. The customers were extremely positive and grateful for the information and were happy to see us out at the grocery stores. I consider the in-store coalition a success because we were able to reach different populations at the grocery store than through our other outreach channels.

We primarily faced two challenges with our outreach efforts as part of this coalition. First, the time when we were out at the stores sometimes did not align with when our target population was shopping. Secondly, some shoppers were hesitant to talk to us because of what seemed to be a point of pride—they did not want to be associated with an assistance program. This is not an uncommon challenge in our outreach efforts, but because the coalition activities take place in such a public location, this challenge may be slightly higher in this setting. However, given that we can reach more and different people, this is a worthwhile trade-off, making our partnership in the coalition well worth our time and effort.

### 3.4. First-Person Account #4: Research Coordinator, UTHealth School of Public Health in El Paso

We joined the In-store coalition to give us an opportunity to connect our chronic disease prevention program, to complementary community resources. We were also drawn to the coalition to offer our program in a grocery store setting while collaborating with other health promotion programs.

The coalition has brought together programs working on similar goals and made it possible for them to implement their work as part of a team. One of the main advantages in our participation has been the opportunity to offer health screenings at a grocery store, which helped us reach more families. We have also gained knowledge about other health promotion programs and services available in the community, particularly the criteria some of them consider for clients to qualify for certain services such as WIC and SNAP. The collaboration with the Heart Smarts program at Food City facilitated our program’s implementation because as we reviewed the participants’ health measures with them, we could refer them to the Heart Smarts program, for nutrition information or if the participants had already seen the nutrition lesson, we could draw from some of the information that they had just received to emphasize the importance of healthy food choices for improving or maintaining health measures overtime.

In our experience at the grocery store, shoppers would enter the store and make their way to the produce section where they would be greeted by the Hearts Smarts team. After speaking with them, Heart Smarts staff would direct people towards our table. The Community Health Workers (CHWs) would then offer the health screenings to the participants. If people were not shopping in a rush, they would at least stop by our table to learn about our program. Often, participants were a little shaken to learn about their health measures (high blood pressure, high BMI, high visceral fat etc.) and how these factors put them at risk of developing chronic diseases, but I think the grocery store setting really helped motivate them to start making healthy lifestyle changes. We noted that many participants who received the health screenings were motivated to apply what they had learned from the Heart Smarts team on healthy eating. For instance, after receiving their screening results, multiple participants looked at the items in their shopping cart and reassessed what they were purchasing.

Challenges we noticed included conflicting schedules that meant we did not get to offer the program in collaboration with some of the other organizations/programs that are part of the coalition. We also noticed after a few weeks that we were seeing the same families and individuals at the store. Since we cannot have duplicated participants within our program, this became a challenge. Another barrier was the lack of privacy to conduct the health screenings. Some participants felt uncomfortable because there was no privacy to obtain their health measures.

Some ways to improve the coalition efforts include involving more of the grocery store managers in the coalition’s plans in order to improve promotion of the programs that are being offered. Additionally, I think having some kind of guide or flyer that describes each coalition member’s programs and how they are tied to the coalition would be very helpful--for all coalition members and potential partners. Finally, it would be great if the coalition could identify additional funding opportunities to support its activities.

### 3.5. First-Person Account #5: Research Associate, The University of Texas at El Paso

The coalition provided a good opportunity to partner with other organizations and reach more venues to offer the services of our project. The biggest advantage to joining the coalition has been that it is easier to establish a relationship with the store managers and secure a spot to offer our services on-site. Thanks to the coalition we were able to set up a booth inside the stores and increase the number of participants we served. We also learned more about the services being offered by other partners at the site.

Compared to when we offer our services in other venues such as health fairs, when we coordinate with the coalition partners in grocery stores, we encourage all of our participants to visit the tables from our partners. Similarly, people who approach our partners first are encouraged to participate with us as well.

We have experienced a positive reception from grocery store customers. They are grateful that we bring health screenings to them. Several of our participants do not have health insurance and haven’t visited a doctor within the last 12 months. We provide them with their health screening results and provide them a referral when a test result indicates they are at increased risk for a health problem. They seem interested in learning more about recipes for healthier diets using low-cost foods.

The coordinated efforts of the coalition partners have been very effective at reaching out to vulnerable populations (e.g., uninsured, and low-income). Many people shopping at the locations targeted by the coalition partners are not aware of their health status. When we provide them with their health screening results, they often want to take action by approaching the other coalition partners to receive more information.

While the coalition has successfully reached a high-need population that will benefit the most from the services each of our partners offer, there is still room for improvement. We need better coordination, regular communication among partners, and clear agreement on collective goals and shared outcome measures. One example where coordination broke down was when there was a delay hearing back from a store manager to get permission to deliver coalition programs and outreach. Some partners moved on to other venues before coordination could be completed. In addition, there were days when some coalition members would not arrive at the venue as planned. It seems we need one centralized coordinating entity or partner who can lead communication with store managers and among coalition partners.

Language and cultural appropriateness could have been a barrier, but fortunately, many of our partners and participants are from a Latino/Hispanic background, and that is an asset. In the grocery store, I have noticed that participants are more open when approached in their first language. For instance, when people approach our booth, we greet them in English and Spanish, and continue the conversation in the language they are most comfortable with continue the conversation in the most comfortable language they are comfortable with. Also, I like that most of the recipes offered by our partners (Heart Smarts) are culturally appropriate for our region.

### 3.6. First-Person Account #6: Outreach and Enrollment Manager, Centro San Vicente Health Center

Before this coalition formed, our team was providing benefits enrollment support in grocery stores. When the coalition first came together, we thought it was a great opportunity to partner with other organizations that shared similar goals and served the local community. One of the biggest advantages of joining the in-store coalition is that we have created a great partnership with these organizations and expanded our outreach to the community. Also, this partnership has helped us learn from the other programs in the coalition and share various ideas on how to improve our program.

Compared to when we were implementing our program separately, the coalition has helped us reach a larger population and increase engagement. For instance, in the days my staff were in the stores with other coalition partners, they reported shoppers were excited about the on-site delivery of the programs and engaged with different services offered by coalition members.

The coalition has been extremely effective at increasing the number of people reached. For example, outreach efforts focused on WIC and SNAP, which are part of this coalition, benefit the low-income population in the region. The coalition has provided a new way to reach this population effectively. Other programs in the coalition, such as Heart Smarts, focus on educating shoppers about how to select and prepare healthy foods which help our community members use their WIC or SNAP benefits to change their eating habits for better health outcomes.

## 4. Discussion

The first-person accounts narrated how and why coalition partners joined the IPOC. Most narratives noted the primary reason was to partner with community agencies that shared common goals and visions. For example, account #4 wrote, “We joined the In-store coalition to give us an opportunity to connect our program, Healthy Fit, to additional community resources that complement or resonate with our program”. This statement concurs with the CCAT formation phase, where partners with similar goals and aspirations collaborate to form a partnership [19]. 

All narratives reported various activities and operations they undertook in the grocery stores, including health screenings, WIC enrollment, SNAP enrollment, and nutrition education, as well as cross-referring shoppers to each other, indicating coordinated efforts within the coalition. These actions resonate with the CCAT maintenance phase, which focuses on the operations and activities of coalitions [19]. Several partners described how the coalition had increased access to a broader population, in part through engagement with grocery stores. For example, #6 noted, “Compared to when we were implementing our program separately, the coalition has helped us reach a larger population and increase engagement”. The primary outcome of the IPOC was to increase people reached with coalition member’s services, which aligns with the institutionalization phase [19].

The narratives also provide perspectives on the strengths, challenges, and recommendations for the IPOC and the grocery store as an intervention setting. Such factors may be especially relevant to others seeking to partner with grocery stores. We discuss these perspectives below.

### 4.1. Coalition Specific Strengths

#### 4.1.1. Synergistic Cooperation

Sharing and pooling resources helped the coalition effectively and efficiently implement activities [30]. Several coalition partners noted that the pooling of resources and access to additional resources were some of the main reasons that motivated members to participate in the coalition. For example, account #4 noted that joining the coalition connected their program to additional community resources that complemented or resonated with their goals. In addition, coalition partners shared how the coalition contributed to strengthening their programs. For instance, account #4 noted, “The collaboration … benefited our program’s implementation …, we could draw from … information that they [the shoppers] had just received [from other coalition partners] to emphasize the importance of healthy food choices for improving or maintaining health measures over time”. As observed from these narratives, complimenting what already existed contributed to strengthening service delivery and reinforcing prior service with the limited resources. 

#### 4.1.2. Passion for the Community

Self-motivation and passion for community change are drivers of public health success [31,32]. Several coalition partners expressed how passion for changing the community’s health contributed to them deciding to join the coalition. For example, account #2 notes they decided to partner with the coalition members driven by their passion for the community, “We wanted to create a healthier community… to improve the health or well-being of people”.

#### 4.1.3. Networking, Coordination, and Collaboration

One of the key foundations of coalitions is establishing partnerships and collaboration toward a shared vision or common goal [22]. As Himmelman [21] notes, coordination includes networking and sharing information about each other’s organizations for mutual benefit and modifying activities for mutual benefit.

The majority of narratives pointed out that the coalition helped them establish partnerships with each other. For example, account #3 stated how they had succeeded in creating stronger partnerships within the collaborating agencies. Account #6 noted, “One of the biggest advantages … is that we have created a great partnership with these organizations and expanded our outreach to the community”. These two examples show how the partners believe that the coalition has expanded their services to the community and led to mutual benefits such as shared resources.

#### 4.1.4. Capacity Building

Most narratives reported that the coalition had created an avenue for self-empowerment and sharing new ideas and knowledge [33]. For instance, account #2 noted how their organization benefited from the continued input, feedback, and learning from other coalition members, “… this coalition has increased our understanding of integral operations of grocery stores …” Also, account #6 noted how the coalition has made it possible to share ideas to improve their program. According to the narratives, the coalition partners have built their capacity with other partnering agencies and shared thoughts on expanding and strengthening their service delivery. Many scholars have reported that internal coalition empowerment and technical assistance are effective ways to enhance coalition functioning and implementation [34,35,36]. 

#### 4.1.5. Culture and Language

Linguistic and cultural skills of bilingual professionals improve program delivery and increase the acceptability of the services offered [37]. The majority of coalition members are bilingual (Spanish and English) and culturally adept in the region; the importance of this was apparent in the accounts. For example, as account #5 wrote, the IPOC took advantage of bilingual staff to increase engagement with the local community and disseminate information in a culturally appropriate and acceptable manner. As evidenced, communicating in a local language, especially to communities with limited English understanding, could help increase community trust and participation [38]. However, account #1 adds that some of the recipes used did not resonate with the older customers and thus suggests coalition partners identify recipes that could meet the “needs of this specific clientele”. These narrations show the importance of recognizing different cultural backgrounds and the fundamental role this plays in collaborative programs [39].

### 4.2. Grocery Store Specific Strengths

#### 4.2.1. Outreach

Program location affects how a program will be received and, therefore, the impact on targeted behaviors [40]. As described in the narratives, most coalition partners were motivated to join other partners in the grocery stores to increase their reach. The partnership was successful in this regard, as most partners reported increasing the number of clients enrolled in their program or using their services through grocery store outreach. For example, account #4 noted, “…our participation has [had] the opportunity to offer health screenings at a grocery store, which helped us reach more families”. Account #6 also adds that “The coalition has been extremely effective at increasing the number of people reached … The coalition has provided a new way to reach this population effectively”.

#### 4.2.2. Lift in Sales

The IPOC had some positive effects on sales and purchases, especially on the food items displayed by the nutrition education team. For example, account #1 noted that the grocery store experienced an increase in the purchase of various food items, “… we see an increase in sales of items used in the recipe demonstrations …” This quote demonstrates how the coalition presence in the grocery stores impacted purchasing of healthy food choices. Also, the narrative indicates how private businesses can benefit symbiotically through coalitions, i.e., contributing to community well-being and simultaneously increasing profits. Similar findings have been reported in other grocery interventions that have increased the purchase and availability of healthy food in low-income neighborhoods [10].

#### 4.2.3. Cross-Referrals

One of the most significant benefits of this coalition was to increase the number of people reached by each program through cross-referral. As accounts #2, #3, and #4 indicate, through the coalition’s efforts at the grocery store, they increased the shoppers exposed to their program. The coalition partners reported having cross-referred shoppers to services offered at the grocery store. For example, account #4 narrates, “In our experience at the grocery store, shoppers would enter the store and make their way to the produce section where they would be greeted by the Hearts Smarts team. After speaking with them, Heart Smarts staff would direct people towards our table. If people were not shopping in a rush, they would at least stop by our table to learn about our program”.

#### 4.2.4. Healthy Food Choices

In addition to increasing access to healthy food through WIC and SNAP enrollment, a main goal of the IPOC was to promote the selection of healthy choices at the grocery stores. The narratives described how having multiple partners at the grocery store influenced healthy food selection. For instance, account #4 noted, “Often, participants were a little shaken to learn about their health status … For instance, after receiving their screening results, multiple participants looked at the items in their shopping cart and reassessed what they should be purchasing”. And the account mentions that “… many participants who received the health screenings were motivated to apply what they had learned from the Heart Smarts team on healthy eating”. This testimony indicates how a coordinated approach (health screening and nutrition education) can influence an individual’s food choices at the point of purchase [41].

### 4.3. IPOC Challenges and Recommendations

#### 4.3.1. Coordination and Conflicting Schedules and Timing

IPOC partners reported the need for coordination as a significant challenge. Account #2 expressed concern about how changes within organizations affected the coalition’s leadership and coordination. Also, account #4 indicated they were not always able to offer their program with other partners due to differences in timing and work schedules. The partners suggested a leadership position be created to coordinate the delivery of services in partnership with all stakeholders. For example, account #5 stated, “We need one centralized coordinating entity or partner who can lead communication with store managers and among coalition partners”. This leader could coordinate with store managers and organize schedules to ensure the coalition members deliver programming simultaneously at the same location. 

#### 4.3.2. Internal Communication

The IPOC members raised concerns regarding internal communication especially in coordinating services at the grocery store. For example, account #4 noted that many partners had limited information about other services offered by IPOC organizations at the grocery store. Thus, the partners recommended the IPOC create a factsheet or flyer that clearly describes each partner’s services, “… some kind of guide that describes each coalition member’s programs … would be very helpful”. Additionally, another partner (account #2) suggested increasing coordination, adding recipes to the grocery store weekly newsletters, and providing better signage to direct shoppers to the coalition activities. Chaskin [15] support the idea of internal communication through sharing knowledge and empowering coalitions which in turn builds capacity at multiple ecological levels (individual, organizational and societal level) and ultimately improves service delivery.

### 4.4. Grocery Store Specific Challenges and Recommendations

#### 4.4.1. Repeated Exposure

Repeated exposure to the same shoppers was one challenge for partners. For example, account #4 noted that they saw the same families and individuals at the store, which hindered them from meeting their recruitment and service delivery targets. “We noticed after a few weeks that we were seeing the same families and individuals at the store. Since we cannot have duplicated participants within our program, [this] became a challenge”. Of note, while this was a challenge for some programs, it was a benefit to others. For example, the Heart Smart program delivers 8 unique nutrition lessons. Repeated contacts with the same shoppers for this program increase shopper exposure to diverse nutrition education lessons.

To address this repeated exposure challenge and expand the coalition’s services, the partners recommended increasing the coalition’s services to other grocery stores in the El Paso region. For example, account #2 noted there were many stores interested in hosting the coalition partners, so it could be beneficial for the coalition to expand its capacity and reach. Also, account #4 proposed that the IPOC reach out to other organizations to increase its partnership and improve marketing strategies.

#### 4.4.2. Space

Lack of sufficient space and privacy in the grocery stores was another challenge. For example, account #6 stated, “Some store locations have limited space, so offering outreach and services from all coalition members at the same time is not feasible”. Similarly, it could have been more effective for programs such as health screening and WIC to have private space. For example, as account #3 indicated, “Some shoppers were hesitant to talk to us because of what seemed to be a point of pride—they did not want to be associated with an assistance program”. The partners, therefore, recommended that the IPOC work with grocery store owners to identify a space within the grocery store that could provide some privacy for programs such as WIC and SNAP.

### 4.5. Limitations and Strengths

Several limitations to this study are important to note. First, the study focuses on one coalition. Findings may not be generalizable to other coalitions and settings dissimilar to IPOC. Also, the evaluation did not study individual program participants. It would be beneficial for future studies to explore shoppers’ perspectives to gain insight into additional ways to enhance the coalition’s impact.

Despite these limitations, this evaluation study reveals the impact that coordinated outreach and programming efforts can have, and does so from first-person accounts of the coalition partners. The coalition approach including grocery stores is novel and could be applied across a diverse array of communities. Therefore, these findings can inform community leaders, organizations, health professionals, and academic partnerships considering strategies to improve community health promotion.

## 5. Conclusions

As the CCAT describes, coalitions take three phases: formation, maintenance, and institutionalization [23]. The IPOC members operationalized the theory through its processes and activities. The most frequently cited success in the coalition was having a positive partnership and collaboration (maintenance phase). Although we cannot numerically quantify the impact of the IPOC, partners’ narratives suggest that the coalition benefitted each participating program. Particularly, the coalition partners demonstrated the suitability, benefits, and opportunities for implementing coordinated health programs in grocery stores. The coalition also proposed recommendations for improving service delivery at the coalition level such as establishing a leadership position or organization that could drive the coalition into the sustainable institutionalization phase [23]. These recommendations will serve the IPOC’s work in new retail locations and provide a critical foundation for efforts to operationalize it more effectively. The challenges and advantages of collaborations within the grocery store settings in IPOC may serve as a useful guide for other communities interested in this type of approach to coalitions.

## Figures and Tables

**Table 1:** IPOC Member organizations and contributions.

Organizations	Sector	Contribution
Special Supplemental Nutrition Program for Women, Infants, and Children (WIC)	Government (Public Health)	WIC enrollment, outreach, assistance
Centro San Vicente (CSV)	Healthcare	SNAP/Medicare/Medicaid enrollment, outreach, assistance
Texas A&M University: Colonias Program and AgriLife Extension	Academic/ Extension	“Shop Heart Smart” ^1^ nutrition education
Food City ^2^	Grocery	Access to three (3) store locations
UTEP & UTHealth	Academic	Health screenings funded by El Paso Department of Public Health
UTHealth CCHI	Academic	Coalition convener, evaluator, trainer

^1^ Shop Heart Smart is a curriculum developed by The Food Trust. UTHealth CCHI worked with The Food Trust to adapt and translate the curriculum into Spanish for delivery in the Paso del Norte region, and provided training for health educators through Texas A&M AgriLife and Colonias Program. ^2^ Food City is a local family-owned grocery retail business with three locations in El Paso.

**Table 2:** IPOC Member Reflection Prompts.

Prompts
Why did you decide to participate in the in-store coalition? What has been the biggest advantage to participating in this coalition? How has the coalition impacted your program? (Probe: goals, knowledge, capacity, technical assistance, etc.) What is different when you implement your program separately from the coalition compared to in coordination with the coalition? Narrate or give your personal experiences with clients/customers and how they received the in-store coalition program? How effective has the coalition been? (Include some examples where possible) Do you consider the in-store coalition a success? Why and/or why not? What are some of the challenges you have experienced in the in-store coalition? What are some of the barriers you might have witnessed with shoppers (e.g., stigma, discrimination, cultural issues etc.)? What could have been done better? Do you have any recommendations for this coalition? What else should we know about the impact of this coalition?

## Data Availability

Not applicable.
